# Single‐cell multi‐modal chromatin profiles revealing epigenetic regulations of cells in hepatocellular carcinoma

**DOI:** 10.1002/ctm2.70000

**Published:** 2024-08-29

**Authors:** Chunqing Wang, Waidong Huang, Yu Zhong, Xuanxuan Zou, Shang Liu, Jie Li, Yunfan Sun, Kaiqian Zhou, Xi Chen, Zihao Li, Shanshan Wang, Yaling Huang, Yinqi Bai, Jianhua Yin, Xin Jin, Shiping Liu, Yue Yuan, Qiuting Deng, Miaomiao Jiang, Chuanyu Liu, Longqi Liu, Xun Xu, Liang Wu

**Affiliations:** ^1^ College of Life Sciences University of Chinese Academy of Sciences Beijing China; ^2^ BGI Research Chongqing China; ^3^ BGI Research Shenzhen China; ^4^ Department of Medical Laboratory Hubei Provincial Clinical Research Center for Parkinson's Disease Xiangyang No.1 People's Hospital, Hubei University of Medicine Xiangyang China; ^5^ Department of Liver Surgery & Transplantation, Liver Cancer Institute, Zhongshan Hospital, Fudan University, and Key Laboratory of Carcinogenesis and Cancer Invasion Ministry of Education Shanghai China; ^6^ Zhongshan‐BGI Precision Medical Center Zhongshan Hospital, Fudan University Shanghai China; ^7^ School of Biology and Biological Engineering South China University of Technology Guangzhou China; ^8^ BGI Research Hangzhou China; ^9^ Shanxi Medical University‐BGI Collaborative Center for Future Medicine Shanxi Medical University Taiyuan China

**Keywords:** chromatin accessibility, epigenomics, hepatocellular carcinoma, histone modifications, multi‐omics, single‐cell sequencing

## Abstract

**Background:**

Various epigenetic regulations systematically govern gene expression in cells involving various biological processes. Dysregulation of the epigenome leads to aberrant transcriptional programs and subsequently results in diseases, such as cancer. Therefore, comprehensive profiling epigenomics is essential for exploring the mechanisms underlying gene expression regulation during development and disease.

**Methods:**

In this study, we developed single‐cell chromatin proteins and accessibility tagmentation (scCPA‐Tag), a multi‐modal single‐cell epigenetic profile capturing technique based on barcoded Tn5 transposases and a droplet microfluidics platform. scCPA‐Tag enables the simultaneous capture of DNA profiles of histone modification and chromatin accessibility in the same cell.

**Results:**

By applying scCPA‐Tag to K562 cells and a hepatocellular carcinoma (HCC) sample, we found that the silence of several chromatin‐accessible genes can be attributed to lysine‐27‐trimethylation of the histone H3 tail (H3K27me3) modification. We characterized the epigenetic features of the tumour cells and different immune cell types in the HCC tumour tissue by scCPA‐Tag. Besides, a tumour cell subtype (C2) with more aggressive features was identified and characterized by high chromatin accessibility and a lower abundance of H3K27me3 on tumour‐promoting genes.

**Conclusions:**

Our multi‐modal scCPA‐Tag provides a comprehensive approach for exploring the epigenetic landscapes of heterogeneous cell types and revealing the mechanisms of gene expression regulation during developmental and pathological processes at the single‐cell level.

**Highlights:**

- scCPA‐Tag offers a highly efficient and high throughput technique to simultaneously profile histone modification and chromatin accessibility within a single cell.
- scCPA‐Tag enables to uncover multiple epigenetic modification features of cellular compositions within tumor tissues.
- scCPA‐Tag facilitates the exploration of the epigenetic landscapes of heterogeneous cell types and provides the mechanisms governing gene expression regulation.

## Graphical abstract

scCPA‐Tag Reveals Multidimensional Epigenetic Heterogeneity.

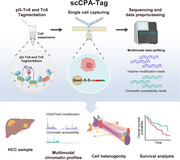

## 1 INTRODUCTION

The regulation of gene expression is orchestrated by a complex interplay of chromatin states, including transcription factors (TFs) binding, *cis*‐acting elements exposure, histone modifications, and chromatin state maintenance by three‐dimensional conformations.^1^ Single‐cell epigenetic sequencing technology is emerging as an essential tool for studying cellular plasticity and heterogeneity.^2^ Profiling changes in chromatin state at the single‐cell resolution facilitates the exploration of pivotal regulatory mechanisms of highly complex and heterogeneous biological systems.^3^, ^4^ Several promising single‐cell sequencing techniques have been developed to capture the individual modality of epigenetic regulations, such as single‐cell assay for transposase‐accessible chromatin using sequencing (scATAC‐seq),^5^ single‐cell Chromatin Proteins and Accessibility Tagmentation (scCUT&Tag),^6^ and single‐cell Droplet‐based bisulfite sequencing  (Drop‐BS).^7^ Many studies have demonstrated limitations in accurately predicting gene regulation and cell fate using single‐dimensional epigenetic data, such as chromatin accessibility or histone modification data.^8^ However, integrating multi‐dimensional epigenomics data from different cells relies on establishing corresponding features across modalities, which presents certain limitations. In recent years, single‐cell multi‐dimensional epigenomics techniques have been developed to explore chromatin accessibility and histone modification in the same cell, such as CUT&Tag2for1,^9^ Single‐cell genome and epigenome by transposase sequencing (scGET‐seq),^10^ and nano‐CUT&Tag (nano‐CT).^11^ However, CUT&Tag2for1 poses challenges in assigning chromatin accessibility and histone modification data based on the distribution of fragment size averages.^9^ scGET‐seq is limited to depicting only H3K9me3 histone modification.^10^ nano‐CT using homemade nanobody‐Tn5 fusion proteins is limited by challenging experimental setups.^11^ Therefore, the emergence of compatible and easy‐to‐implement single‐cell multi‐dimensional epigenomics techniques is needed.

Here, we developed an effective high‐throughput single‐cell multi‐modal epigenetic approach named single‐cell Chromatin Proteins and Accessibility Tagmentation (scCPA‐Tag), to comprehensively capture the landscapes of histone modification/TFs binding along with chromatin‐accessible regions at the single‐cell level. scCPA‐Tag utilizes a fusion protein G to Tn5 transposase (pG‐Tn5) and a Tn5 transposase with unique barcodes, which allows specific assignment of the histone modification and chromatin accessibility profiles in the same cell. scCPA‐Tag is compatible with profiling different histone modification marks. scCPA‐Tag presents a comparable sensitivity, specificity, and repeatability compared with ATAC‐seq and CUT&Tag assessed by the K562 cell line. We applied scCPA‐Tag to a hepatocellular carcinoma (HCC) sample and revealed distinct epigenetic features of heterogeneous cell types and tumour cell subsets in HCC. The application of scCPA‐Tag provides insights into how gene expression and transcription start site (TSS) selection are regulated by chromatin accessibility and histone modifications, for example explaining the regulation mechanism for different isoform usage of *ACTA2* between fibroblasts and lymphocytes. A more aggressive tumour cell subtype (C2) was identified and characterized by high chromatin accessibility and low H3K27me3 on genes associated with cell proliferation, invasion, and metastasis using scCPA‐Tag. Altogether, our study demonstrates that scCPA‐Tag enables the mapping of histone modification binding DNA and chromatin‐accessible regions at the single‐cell level, facilitating epigenetic investigations of cell fate determination and disease‐related cellular heterogeneity.

## 2 METHODS

### 2.1 Clinical sample and single‐cell isolation

The fresh tumour sample was obtained from a 47‐year‐old male patient diagnosed with hepatocellular carcinoma (HCC) at Zhongshan Hospital, Fudan University. This HCC patient was diagnosed with Barcelona Clinic Liver Cancer classification (BCLC) stage C, hepatitis B virus (HBV) infection, alpha‐fetoprotein (AFP) level of 9 ng/mL, and underwent transarterial chemoembolization (TACE) treatment.

The fresh tumour specimen was surgically excised from the patient and placed in 90% Dulbecco's modified eagle medium and 10% fetal bovine serum. Following that, the tissue blocks were cut into small pieces 1−3 mm in diameter within a refrigerated container. These pieces were then transferred to the gentle MACS C tubes along with 5 mL of digestive enzyme provided in the MiltenyiBiotec tumour dissociation kit (# 130‐095‐929). Subsequently, the tissues underwent dissociation to obtain a single‐cell suspension using the gentleMACS Dissociator. Finally, the suspension was subjected to centrifugation at 400×*g* for 7 min, resuspended in CELLSAVING solution, and then stored at −80°C.

### 2.2 K562 cell line

The K562 cell line consisted of cultured cells of myeloid leukaemia, procured from the American Type Culture Collection. These cells were cultured in RPMI‐1640 supplemented with 15% fetal bovine serum and 1× Penicillin–Streptomycin antibiotic.

### 2.3 pG‐Tn5 and Tn5 transposase embedding

Primer A, primer B1‐B16, and primer C1‐C2 were dissolved to 100 µM using an annealing buffer (10 mM Tris‐HCl, 1 mM EDTA, and 50 mM NaCl). We mixed equal amounts of primer B1–B8 (Table S1) as primer B mix1, and mixed equal amounts of primer B9‐16 as primer B mix2. Then, we mixed 10 µL primer A with 10 µL primer B mix1 as adapter AB1, 10 µL primer A with 10 µL primer B mix2 as adapter AB2, 10 µL primer A with 10 µL primer C1 as adapter AC1, and 10 µL primer A with 10 µL primer C2 as adapter AC2. The adapters were placed in a thermal cycler with a heated lid set at 105°C using the following conditions: 72°C for 15 min, 60°C for 10 min, 50°C for 10 min, 40°C for 10 min, and 25°C for 30 min. When the reaction was complete, the adapter AB1 was mixed with AC1 and named as adapter 1, the adapter AB2 was mixed with AC2 and named as adapter 2. 1.4 µL of adapter 1, 8 µL of pG‐Tn5 transposase (500 ng/µL), and 5.6 µL of coupling buffer (Vazyme S602 kit) was prepared. They were gently mixed with the pipette, and the mixture was incubated in a cycler at 30°C for 1 h with a heated lid set at 50°C. 6.25 µL of adapter 2 and 43.75 µL of Tn5 transposase (1 U/µL) were prepared, mixed gently with the pipette, and incubated in a cycler at 25°C for 1 h with a heated lid set at 50°C. Adaptor‐loaded pG‐Tn5 and Tn5 transposase were preserved at −20°C.

### 2.4 CPA‐Tag antibody incubation and tagmentation

A total of 300,000 K562 cells were resuspended in 1 mL cell wash buffer (containing 1× Protease inhibitor cocktail; 150 mM NaCl, 20 mM pH 7.5 HEPES, and 0.5 mM Spermidine). The cells were then pelleted at 500×*g* for 5 min at 4°C and the supernatant was discarded. Cells were resuspended in 1 mL permeability buffer (containing 0.01% Digitonin and 0.01% NP40 in wash buffer) to change the permeability of cells. Then, we added 100 µL permeability buffer with 2 mM EDTA and added 1%−2% primary antibody (H3K27me3 or H3K4me1) into the cell pellet. The mixture was incubated overnight on the shaker at 4°C. Cells were washed with permeability buffer and were pelleted at 500×*g* for 5 min at 4°C. Cells then were incubated at 4°C for 1 h with the secondary antibody (Guinea Pig anti‐Rabbit IgG [Heavy & Light Chain] Antibody, ANTIBODIE) diluted 1:50 in 100 µL permeability buffer. After that, cells were washed three times with permeability buffer and pelleted at 500×*g* for 5 min at 4°C. A 1:100 dilution of pG‐Tn5 transposase was prepared in pre‐tagmentation buffer (0.01% NP40, 0.01% Digitonin, 20 mM pH 7.5 HEPES, 300 mM NaCl, 0.5 mM Spermidine, and 1× Protease inhibitor cocktail), and resuspended 60,000 cells with 100 µL mix reagent, and then incubated 1 h at 4°C on the shaker. After that, cells were washed 3 times with pre‐tagmentation buffer and cells were pelleted at 300×*g* for 6 min at 4°C. After removing the supernatant, cells were resuspended in 100 µL tagmentation buffer (10 mM MgCl_2_ in pre‐tagmentation buffer) and incubated for 1 h at 37°C. Next, pelleted cells at 300×*g* for 6 min at 4°C, and then accessible chromatins regions were cleaved and tagged by 25 µL Tn5 transposase mix (5 µL Tagmentation buffer, 4 µL transposase, 16 µL 1% BSA/PBS) for 30 min at 37°C. DAPI‐stained cells were visualized under the microscope to detect whether the cells were clumping and count the number of cells.

When performing scCUT&Tag, we just need to use pG‐Tn5 to transpose chromatin and skip the traditional Tn5 transpose, and then perform droplet generation. When we just want to perform a traditional scATAC‐seq, we skip the antibody incubation and pG‐Tn5 transposing step.

### 2.5 scCPA‐Tag library construction

Single‐cell capture was performed using the DNBelab C Series system Single‐Cell ATAC Library Prep Set (MGI, 1000021878).^12^, ^13^ In brief, 10,000 transposed single cells suspensions (20 µL cells, 8 µL ATAC Enzyme I, 46.7 µL ATAC nuclei Buffer, 25.3 µL Nuclease‐Free Water) and 100 µL beads suspension (300,000 beads, 40 µL Additive C, 58 µL ATAC Bead buffer, 2 µL Tn primer, 183+C1 primer and 283+C2 primer) (Table S1) were prepared for per chip to generate droplets. After droplet generation, the droplets were collected and performed pre‐amplification in the droplet following the procedure in a cycler: 72°C for 30 min, 98°C for 30 s, 10 cycles of 98°C for 10 s, 63°C for 30 s and 72°C for 5 s, final extension at 72°C for 1 min with the heated lid set at 105°C. After droplet pre‐amplification, we performed emulsion breakage and captured beads collection. Next, resuspend beads with 400 µL ATAC Enzyme III, 32 µL Tn primer and phosphorylated 183 primer, 133.6 µL Additive E, and 234.4 µL nuclease‐free water. Then this mixture was divided into eight PCR tubes to amplify the DNA with histone modification. The PCR amplification program was set at 98°C for 30 s, 18 cycles of 98°C for 10 s, 63°C for 30 s, and 72°C for 5 s, final extension at 72°C for 1 min with the heated lid set at 105°C.

The cell beads and PCR supernatant were split, and the supernatant was purified with VAHTS DNA clean beads using 0.5× and 0.5× two‐side purification according to the DNBelab C Series system single‐cell ATAC library Prep Set. The cell beads were used for amplification of chromatin‐accessible DNA with 400 µL ATAC Enzyme III, 32 µL Tn primer and phosphorylated 283 primer, 133.6 µL Additive E, and 234.4 µL nuclease‐free water. The mixture was divided into eight PCR tubes. The PCR amplification program was the same as the above step of amplification for DNA with histone modification. After that, the DNA was purified with 0.5× and 0.5× VAHTS DNA clean beads. Then, the concentrations of DNA were measured using a Qubit dsDNA assay kit, and the Agilent 2100 Bioanalyzer was used to detect fragment size of the DNA. Finally, libraries were sequenced on a BGISEQ‐500 sequencer with 50 bp for read 1, 50 bp for read 2, and 48 bp for barcode.

### 2.6 scCUT&Tag library construction

For constructing scCUT&Tag libraries, cells were incubated with a primary antibody, a second antibody, and pG‐Tn5 transposase. Finally, the cells underwent pG‐Tn5 transposase program and were dyed with DAPI to count 10,000 cells and generate single‐cell droplets. 2 µL Tn primer and 183+C1 primer were added to the beads’ suspension for pre‐amplification. Emulsion breakage and amplification followed the same procedures as amplifying histone‐modified DNA obtained by scCPA‐Tag. Finally, libraries were sequenced on a BGISEQ‐500 sequencer with 50 bp for read 1, 50 bp for read 2, and 26 bp for barcode.

### 2.7 snATAC‐seq library construction

Firstly, the cell nuclei were prepared as the DNBelab C Series system Single‐Cell ATAC library Prep Set. One million K562 cells were resuspended in 100 µL Cell lysis buffer (10 mM Tris‐HCl, 10 mM NaCl, and 3 mM MgCl_2_, 0.1% Tween‐20, 0.1% NP‐40, 0.01% Digitonin, 1% BSA), and incubated on ice for 5 min. Next, we added 1 mL of ATAC wash buffer (10 mM Tris‐HCl, 10 mM NaCl, 3 mM MgCl_2_, 0.1% Tween‐20, 1% BSA) to resuspend the cells and centrifuged them at 500×*g* for 5 min to stop the lysis reaction. We repeated the wash step by resuspending the nuclei in 500 µL of ATAC wash buffer and centrifuging at 500×*g* for 5 min. Finally, the nuclei were resuspended in 50 µL 1× PBS with 1% BSA and stained with DAPI to count the number of nuclei.

Second, snATAC‐seq libraries were constructed using the DNBelab C Series system single‐cell ATAC library prep set. In brief, the processes include nuclei transposition, droplet generation, pre‐amplification, emulsion breaking, DNA amplification, purification, and library quality control. The paired‐end sequencing was conducted on the MGI DNBSEQ‐T1 platform, employing the following read length: 109 bp for read 1, 50 bp for read 2, and 10 bp for the sample index.

### 2.8 Bulk CPA‐Tag library construction

After the pG‐Tn5 and Tn5 transposase tagmentation reaction, the cell mixture was collected and DNA was purified using MiniElute PCR purification kit (Qiagen, cat. 28004) and eluted with 42 µL NF‐H_2_O. Then, 21 µL DNA was mixed with 25 µL 2× Golden High‐Fidelity ReadyMix, 2 µL Bulk N5 primer, and 2 µL phosphorylated 183+C1 primer to construct a histone modification library. Another 21 µL DNA was mixed with 25 µL 2× Golden High‐Fidelity ReadyMix, 2 µL Bulk N5 primer and 2 µL phosphorylated 283+C2 primer to construct a chromatin‐accessible library. The samples were placed in a cycler with a heated lid at 105°C under the following conditions: 72°C for 5 min, 98°C for 30 s, 12 cycles of 98°C for 10 s, 63°C for 30 s and 72°C for 5 s, final extension at 72°C for 1 min and holding at 12°C. The amplified products were purified with 0.5× and 0.5× VAHTS DNA clean beads. Qubit dsDNA Assay kit and the Agilent 2100 Bioanalyzer were used to detect DNA concentration and fragment size. Finally, the DNA was sequenced on a BGISEQ‐500 sequencer with 50 bp for read 1, 50 bp for read 2, and 22 bp for barcode.

### 2.9 Bulk data processing

The original dual‐omics sequencing reads were split using Python with sixteen barcode adapters, then the reads were aligned to the hg19 reference genome by the BWA mem function (v0.7.12).^14^ Using Samtools (v1.9)^15^ to convert the comparison result from sam format to bam format, and removing duplicates through Picard (v2.23.8) (https://broadinstitute.github.io/picard/). For chromatin‐accessible data, using MACS2 (v2.1.1)^16^ for peak calling with parameters: “‐f BAM ‐B ‐q 0.05 –keep‐dup all”. For histone modification data, we used SEACR (v1.4)^17^ to call peaks from the aggregation profile for each sample with the following settings: “‐c 0.05 ‐n non ‐m stringent”. For peak annotation, we used the R package ChIPseeker (v1.30.0).^18^

### 2.10 Single‐cell data processing

The original dual‐omics sequencing reads were split using Python with sixteen barcode adapters, and then the data were converted to Fastq+ by PISA (v0.7).^19^ Reads were aligned to the hg19 reference genome by the BWA mem function.^14^ Using Samtools to convert the comparison result from sam format to bam format, and then using d2c (v1.3.8, https://github.com/STOmics/d2c) (the setting of snATAC‐seq is “–mapq 30 ‐c 10 –sat –bt1 CB”, the setting of scCUT‐seq is “–mapq 30 ‐ ‐bf 500 ‐c 10 –sat –bt1 CB”) for deconvolution to generate fragment files of each library for the following analysis. For chromatin‐accessible data, we use MACS2 for peak calling with parameters: “‐f BAM ‐B ‐q 0.01 –nomodel”. For histone modification data, use SEACR to call peaks from the aggregation profile for each sample with the following settings: “‐c 0.05 ‐n non ‐m stringent”. Annotate peaks using the R package ChIPseeker.

### 2.11 Peak overlap between samples and signal quantification

Firstly, standardize the read counts across samples to ensure uniformity. Subsequently, employ the enrichPeakOverlap function from the ChIPseeker R package to compute the overlapping peaks. To quantify signal intensity at peaks, the number of fragments overlapping peaks were collected via bedtools (v2.30.0)^20^ coverage with the “‐counts” option, and then subsequently converted to counts per million.

### 2.12 Enrichment and library correlation analysis

The metagene plots were generated by using the deeptools (v3.5.1) package,^21^ utilizing the computeMatrix and plotHeatmap scripts. To obtain scores for individual peak regions, the deeptools multiBigwigSummary script was employed. Additionally, correlation heatmap plots were created by using the deepTools plotCorrelation script.

### 2.13 Cell bigwig segmentation

The cell bigwig segmentation utilized the filter barcodes function from sinto (v0.9.0) (https://timoast.github.io/sinto).

### 2.14 Analysis of quality control and clustering

TSS enrichment fraction and fragmentation per single cell were calculated by ArchR (v1.2.0).^22^ If the TSS score of the cell is less than a certain value (histone modification data: 0, chromatin‐accessible data: 2) or the total number of fragments is less than 500, the cell will not participate in the subsequent analysis. ArchR performs dimensionality reduction and clustering using iterative latent semantic indexing (LSI) clustering. Briefly, it creates 500‐bp (histone modification data: 5000‐bp) windows across the entire human genome and quantifies how accessible each cell is within each window. Next, it performs LSI dimensionality reduction using the addIterativeLSI function with parameters “iterations = 2, dimsToUse = 1:30, sampleCells = 10000, varFeatures = 25000”. Clustering is then performed by using Seurat's FindClusters function with parameters “reducedDims = ‘IterativeLSI’, method = ‘Seurat’, resolution = 1”. Gene scores of H3K27me3 and chromatin accessibility were calculated by the addGeneScoreMatrix function with the default parameters, and different gene scores for each cluster were calculated by getMarkerFeatures function using ArchR. The MACS2 was used with peak calling by default parameters on the Tn5‐corrected insertions (each end of the Tn5‐corrected fragments) for each cluster.

### 2.15 Analysis of identifying different features

The marker peaks of each cluster were defined through the getMarkerFeatures function, and the motif enrichment score was calculated by the addMotifAnnotations and peakAnnoEnrichment function in ArchR with default parameters, and the significant different peaks were identified with adjust *p* ≤ 0.05 and Log_2_ (fold change) ≥ 0.5.

### 2.16 Functional enrichment analysis

The gene ontology (GO) term analysis was calculated and performed by ClusterProfiler (v4.2.0).^23^ The terms with a *p*‐value less than 0.05 were considered significantly enriched. The differentially accessible or silent genes of each cluster in chromatin‐accessible data or histone modification data are obtained based on Gene scores from ArchR.

### 2.17 Survival analysis

According to the expression of identified features, the proposed threshold was calculated by the surv_cutpoint function in the survminer (v0.4.9) package (https://github.com/kassambara/survminer), and then the patients were divided into two groups for comparison. The Kaplan–Meier survival curves showed the fractions of patients living in a certain time and assessed the effect of the particular feature on prognoses. Statistical significance was calculated with the log‐rank test.

### 2.18 Statistical analysis

Data analyses were performed using R version 4.2.1 and Python 3.9.12. Detailed descriptions of the statistical methods employed can be found in the corresponding sections of the “Methods” subsections. *p*‐values were computed utilizing the Wilcoxon rank‐sum test and adjusted for false discovery rate in instances of multiple hypothesis testing. Statistical significance was defined as *p* < 0.05. All boxplots show the median and 25th to 75th percentile of data distribution.

## 3 RESULTS

### 3.1 The scCPA‐Tag parallel profiled histone modification and chromatin accessibility at single‐cell resolution

**FIGURE 1:**
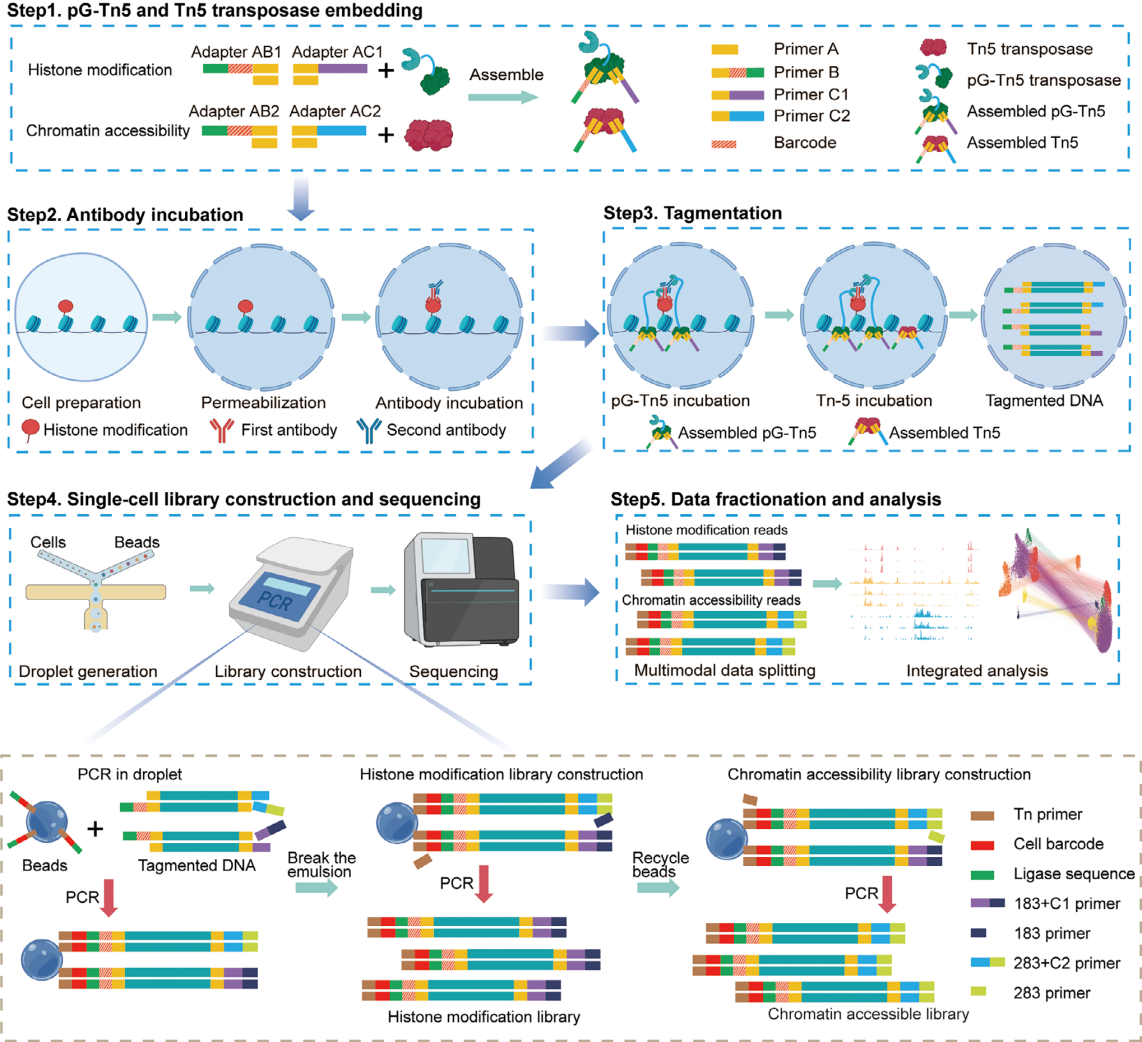
The schematic of the scCPA‐Tag. (1) Embed the pG‐Tn5 transposase and Tn5 transposase with adapters containing different barcodes. (2) Change the permeability of cells and incubate primary antibody and secondary antibody. (3) The pG‐Tn5 and Tn5 sequentially cleave and tag the chromatin DNA surrounding the antibody and the accessible chromatin regions, respectively. (4) Collect the transposed nuclei and generate droplets to capture single cell, construct library and sequence. (5) The sequencing data was split by barcodes on the adapters, allowing for separate analysis of histone modification profiles and chromatin accessibility profiles.

To investigate multiple dimensions of epigenetic information regulating gene expression, we developed a novel technique called scCPA‐Tag. This innovative approach utilizes a pG‐Tn5 and a Tn5 transposase, which are embedded with different adapters and barcodes for labelling the histone modification and chromatin accessibility insertions in individual cells (Figure 1). The process of scCPA‐Tag involves the following steps: (1) embedding pG‐Tn5 transposase and Tn5 transposase with different primer B and primer C sequences of adaptor to identify the information captured by the two transposase; (2) treating cells with a specific antibody to bind to the target protein, followed by adding a secondary antibody to increase binding sites for pG‐Tn5; (3) transposing the chromatin of cells using pG‐Tn5 and Tn5 with different adapters, which these barcodes allow for marking the regions of histone modification and accessible chromatin, respectively; (4) generating single‐cell libraries using a modified the DNBelab C Series system Single‐Cell ATAC protocol to obtain the scCPA‐Tag library and sequencing; (5) pairing profiles from the same single cell using the cell barcode on the beads in the droplet, and then splitting paired two‐dimensional data according to adapters on the Tn5 and pG‐Tn5 (Figure 1, see Section 2 for details).

We performed multi‐modal scCPA‐Tag, unimodal scATAC‐seq, and scCUT&Tag on K562 cells using antibodies against H3K27me3 and H3K4me1, respectively, and included published bulk CUT&Tag^24^ and bulk ATAC‐seq^25^ data of K562 as a reference to assess the performance of scCPA‐Tag. The fragment size distribution of scCPA‐Tag showed a nucleosomal ladder pattern, consistent with results obtained from unimodal control experiments (Figure S1A). The functional annotation of the peak regions identified by the scCPA‐Tag data revealed that peaks associated with chromatin accessibility and H3K4me1 were predominantly enriched in the promoter regions, accounting for approximately 37.25% and 41.26%, respectively. In contrast, H3K27me3 modification showed lower enrichment in promoter regions at around 15.73% (Figure S1B). These consistent trends were also observed in the reference data (Figure S1B).

**FIGURE 2:**
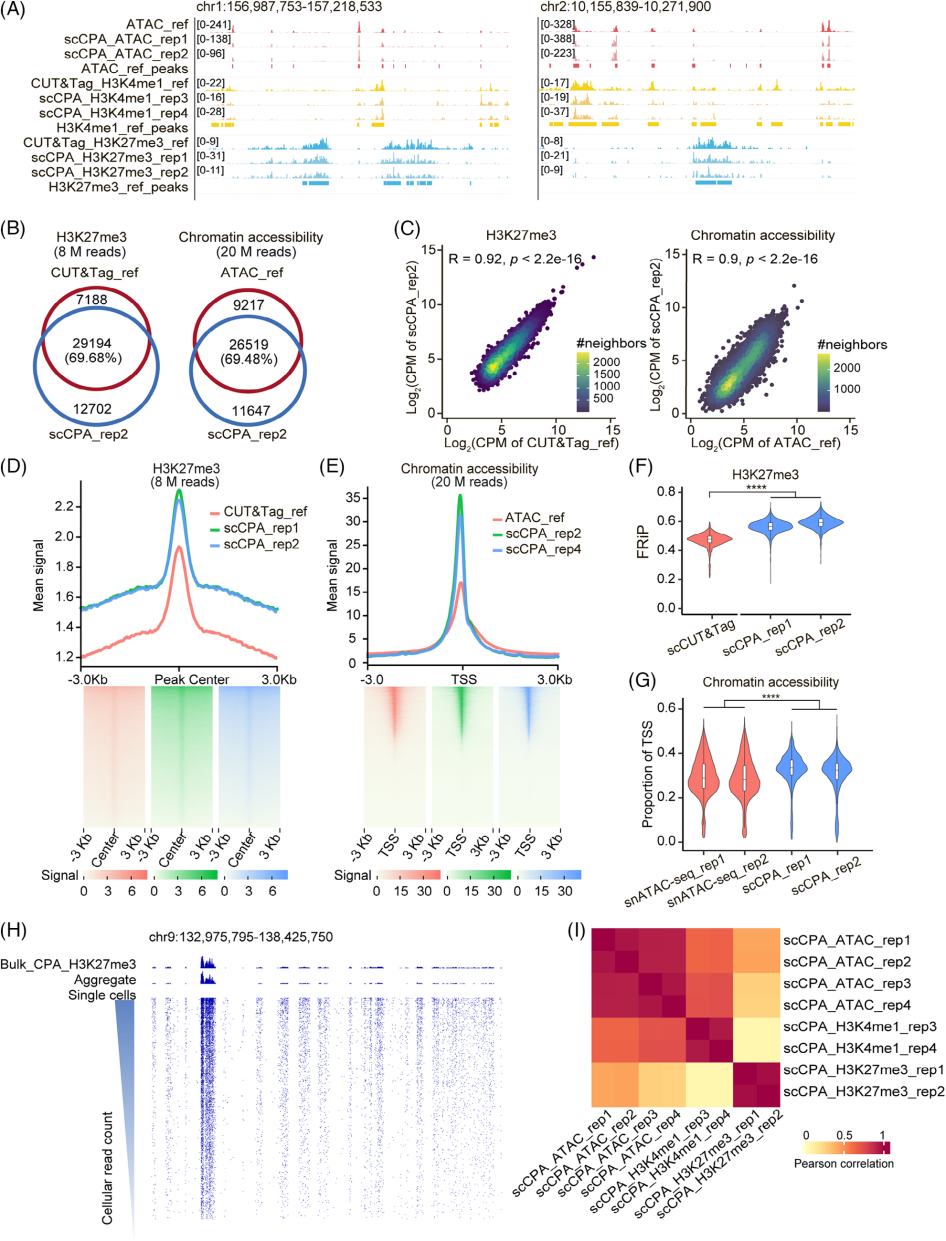
scCPA‐Tag accurately maps the histone modification and chromatin accessibility within individual cell. (A) The genome browser screenshot shows chromatin accessibility (red), H3K4me1 (yellow), and H3K27me3 (blue) signals from ATAC‐seq (row 1), CUT&Tag (rows 5 and 9), and scCPA‐Tag (rows 2, 3, 6, 7, 10, and 11). ref: reference data; rep: replication experiments of scCPA‐Tag. (B) Venn diagrams show the overlap of H3K27me3 peaks identified in scCPA‐Tag and CUT&Tag (left) at downsampled read depths of 8 million reads and the overlap of chromatin accessibility peaks identified in scCPA‐Tag and ATAC‐seq (right) at downsampled read depths of 20 million reads. (C) Density scatter plots display the correlation of peak signals of H3K27me3 and chromatin accessibility between scCPA‐Tag and CUT&Tag, as well as ATAC‐seq, respectively. Each dot represents an individual peak in the unified peak set with a colour scale indicating density. Pearson's correlation value is shown at the top of each plot. CPM: counts per million. (D–E) Signal comparison of H3K27me3 histone modification and chromatin accessibility in CUT&Tag, ATAC‐seq, and scCPA‐Tag in K562 cells. Profiles of the H3K27me3 (D) and chromatin accessibility (E) were compared at downsampled read depths of 8 million and 20 million reads, respectively. (F) Violin plots show the fraction of reads in peaks of H3K27me3 histone modification between scCUT&Tag and scCPA‐Tag. ****p < 0.0001, Wilcoxon rank‐sum test. (G) Violin plots show the proportion of TSS per cell of chromatin accessibility between snATAC‐seq and scCPA‐Tag. ****p < 0.0001, Wilcoxon rank‐sum test. (H) Chromatin segments of the human genome are shown for H3K27me3 of bulk CPA‐Tag and scCPA‐Tag on K562 cells. Single cells are ordered by total read counts in each cell. (I) Heatmap of correlation of scCPA‐Tag profiles replication. The colour scale corresponds to the Pearson correlation value.

The distribution patterns of scCPA‐Tag fragments for histone modification and chromatin accessibility were highly concordant with those identified by unimodal CUT&Tag and ATAC‐seq reference data (Figure 2A; Figure S1C). The majority of peaks detected by scCPA‐Tag overlapped with the corresponding reference data for H3K27me3 (69.68%), H3K4me1 (69.26%), and chromatin accessibility (69.48%) (Figure 2B; Figure S1D). Also, there were strong positive correlations in peak signals between scCPA‐Tag and reference datasets of CUT&Tag and ATAC‐seq (*R* = 0.92, 0.82, 0.90 for H3K27me3, H3K4me1, and chromatin accessibility, *p* < 0.05) (Figure 2C; Figure S1E). Furthermore, the histone modification data obtained from scCPA‐Tag showed comparable signal‐to‐noise ratios to those of the reference data (H3K27me3: 1.47 and 1.43 vs. 1.54; H3K4me1: 1.98 and 1.97 vs. 2.33), while the chromatin accessibility data generated by scCPA‐Tag exhibited higher signal‐to‐noise ratios compared with the reference (24.51 and 20.84 vs. 8.42) at equivalent read depths (Figure 2D,E; Figure S1F). These results demonstrated that scCPA‐Tag accurately and specifically profiles multiple layers of epigenetic information within individual cells.

### 3.2 scCPA‐Tag with high specificity and reproducibility to analyze epigenetic states in the single cell

The scCPA‐Tag enables the capture of two‐dimensional epigenetic information within individual cells with a high throughput of thousands of cells per run by integrating with the droplet‐based microfluidic chip device DNBeLab C system (Table S2). After quality control, scCPA‐Tag generated 1724 fragments of histone modification and 3129 fragments of chromatin accessibility per cell on average (Figure S1G–I, Table S2). The H3K27me3 and H3K4me1 profiles generated by scCPA‐Tag showed a higher fraction of reads in peaks (FRiP) than those obtained from unimodal scCUT&Tag (H3K27me3: 0.53 vs. 0.48 and H3K4me1: 0.48 vs. 0.46, *p* < 0.01; Figure 2F; Figure S1J). Additionally, the proportion of TSS in chromatin accessibility profiles generated by scCPA‐Tag was higher than that observed in unimodal scATAC‐seq (0.31 vs. 0.28; Figure 2G). These results indicate that scCPA‐Tag has comparable specificity for detecting epigenetic profiles. Furthermore, most fragments (85.39% for H3K27me3 and 83.33% for chromatin accessibility) from individual cell aligned with the peaks identified by the bulk profiles, and a high positive correlation was observed between the peaks identified by aggregated pseudo‐bulk and bulk data (*R *= 0.97, *p *< 0.05) (Figure 2H; Figure S2A–C). High reproducibility of scCPA‐Tag across replicates was observed (*R *> 0.9, *p *< 0.05) (Figure 2I).

Therefore, scCPA‐Tag demonstrates remarkable specificity and reproducibility in identifying gene enhancer regions (H3K4me1) or silencer regions (H3K27me3) with accessible chromatin regions in the same cell.

### 3.3 scCPA‐Tag revealed the regulation of gene expression in K562 cells

**FIGURE 3:**
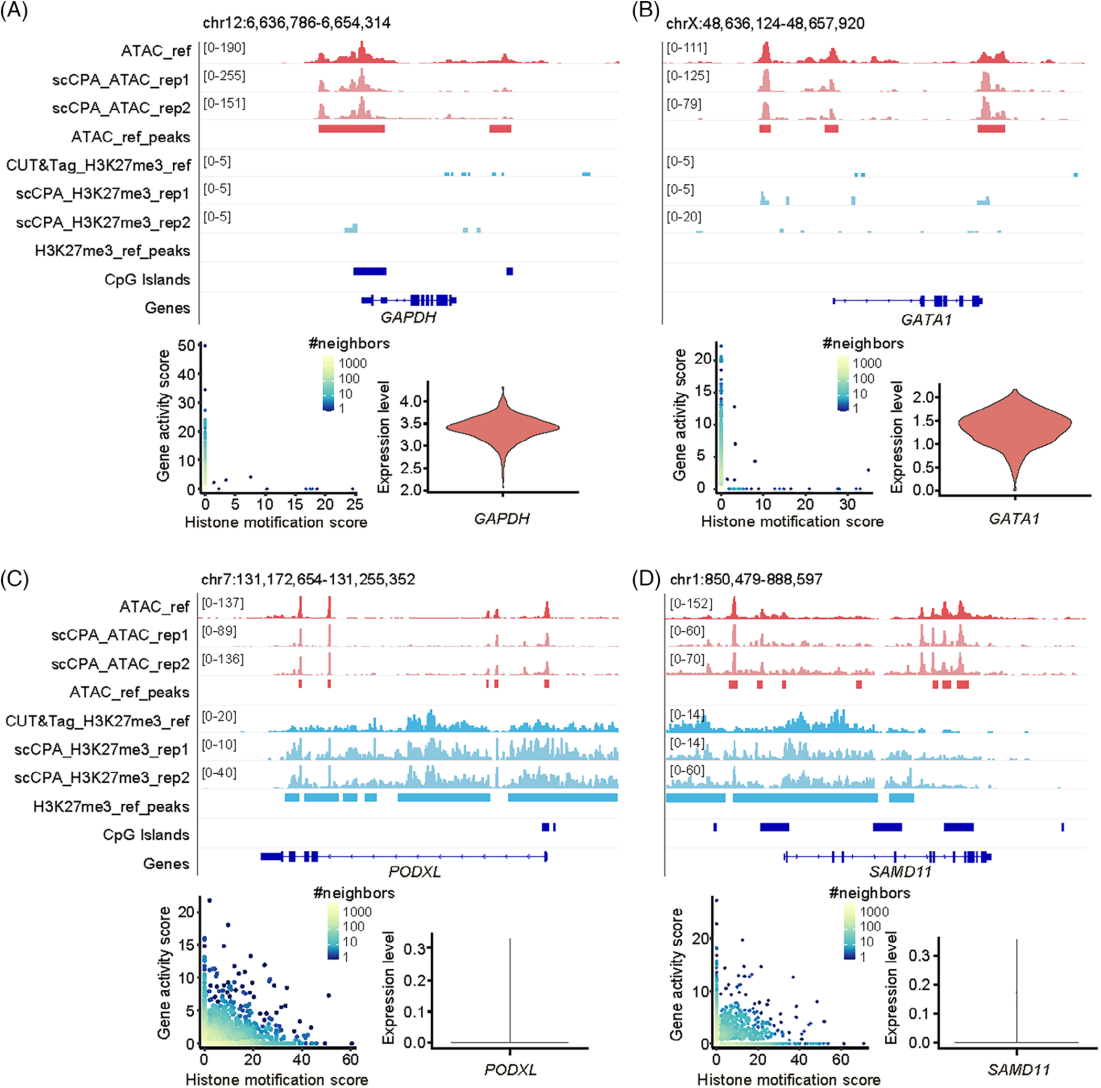
scCPA‐Tag revealed the regulation of gene expression A–D. The upper panels depict the signal of the gene's chromatin accessibility or H3K27me3 histone modification from K562 cells. In the genome browser screenshot, the X‐axis represents the chromatin region of genes, the Y‐axis represents fragment counts, and each row represents a set of experimental data. On the left bottom panel, the density scatter plot shows the degree of H3K27me3 histone modification (X‐axis) and chromatin accessibility (Y‐axis), with each dot representing an individual cell. On the right bottom panel, the violin plot represents the gene expression in K562 cells.

We next investigated the co‐regulation of histone modification and chromatin accessibility on gene expression in K562 cells. The scRNA‐seq data of K562 cells were obtained from the publicly available data.^26^ Specifically, we quantified the levels of H3K27me3 and chromatin accessibility of genes by calculating gene scores using ArchR^22^ to examine the relationship between epigenetic features and gene expression. It was observed that most regions (97.3%) are either chromatin accessible or have H3K27me3 modification in K562, but there are certain regions (2.7%) where accessible chromatin overlapped with H3K27me3 modification (Figure S2D). As expected, genes with accessible chromatin surroundings but with minor or no H3K27me3 modification exhibited high expression levels. This pattern was observed in the housekeeping genes, which are involved in essential cellular functions,^27^ such as *GAPDH* and *ACTB*. Erythroid differentiation transcription factor genes *GATA1* and *KLF1*, involved in the process of erythrocyte cell formation and showing high expression in K562 cells,^28^, ^29^ also exhibited this pattern (Figure 3A,B; Figure S2E,F).

In addition, we observed several genes whose promoter regions exhibited chromatin accessibility and had significant levels of inhibitory H3K27me3 modification, resulting in gene silencing in K562 cells (Figure 3C,D). Examples of such genes include *PODXL*, which encodes an anti‐adhesive glycoprotein and is primarily expressed in epithelial cells of kidney glomeruli,^30^ and *SAMD11*, playing a crucial role in photoreceptor development.^31^ H3K27me3 modification peaks at certain CpG island loci of *PODXL* and *SAMD11* were also observed (Figure 3C,D). The presence of H3K27me3 modification potentially inhibited the expression of these chromatin‐accessible genes in K562 cells, highlighting the crucial role of epigenetic states in maintaining proper cellular functions (Figure 3C,D).

In summary, our results highlighted the significance of multi‐modal epigenetic information for comprehensively revealing mechanisms of gene expression regulation.

### 3.4 scCPA‐Tag revealed the cell heterogeneity in HCC

Epigenetic changes drive aberrant transcriptional programs in tumours. We applied scCPA‐Tag with H3K27me3 antibody in a HCC tumour sample to discriminate heterogeneous cell populations and explore their epigenetic profiles. After quality control (see Methods), 5843 cells from chromatin‐accessible profiles and 9274 cells from H3K27me3 profiles were obtained for subsequent analyses. Similar to the performance in K562 cells, the distribution of fragment size showed a nucleosomal ladder pattern characterized by mono‐, di‐, and tri‐nucleosome periodic changes (Figure S3A). No batch effect was observed between the two libraries of chromatin accessibility and H3K27me3 libraries according to their clustering results (Figure S3B).

**FIGURE 4:**
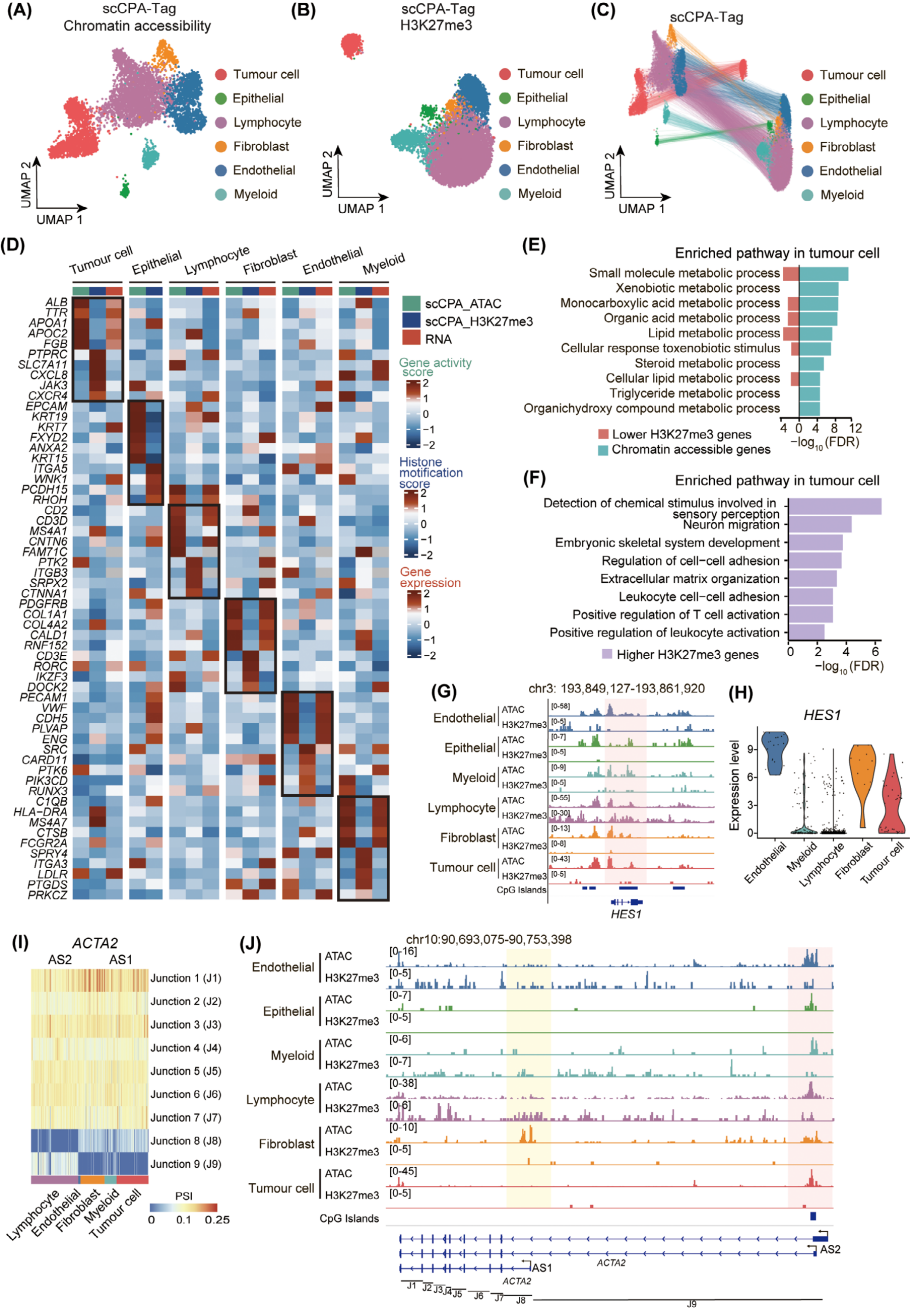
scCPA‐Tag reveals cell heterogeneity and epigenetic regulation in HCC. (A) The UMAP plot shows the annotation and colour codes for cell types in the HCC sample based on chromatin‐accessible libraries generated by scCPA‐Tag. (B) The UMAP plot shows the annotation and colour codes for cell types in the HCC sample based on H3K27me3 modification libraries generated by scCPA‐Tag. (C) UMAP embedding of the scCPA‐Tag data for H3K27me3 modification and chromatin accessibility. The lines connect representations of the same cells in the individual dimensions. (D) Heatmap shows the chromatin accessibility, H3K27me3 modification, and RNA expression of marker genes in each cell type. (E) Two‐sided bar graph shows the pathway enriched in lower H3K27me3 modification genes (red) and chromatin‐accessible genes (cyan) in tumour cells. (F) The bar graph shows the H3K27me3 peaks upregulation associated pathways in tumour cells. (G) Genome browser scCPA‐Tag view of the *HES1* region for H3K27me3 modification and chromatin accessibility profiles in various cell types. (H) Violin plot shows *HES1* expression in various cell types from previously published RNA‐seq data. (I) Heatmap of PSI value. Columns represent cells, rows represent the percent spliced in (PSI) values of different junctions. (J) Genome browser scCPA‐Tag view of the *ACTA2* region for H3K27me3 modification and chromatin accessibility profiles in various cell types. The yellow label TSS region of AS1, the red label TSS region of AS2.

We identified six major cell clusters, including tumour cell, epithelial cell, lymphocyte, fibroblast, endothelial cell, and myeloid cell, by analyzing the accessible chromatin data of scCPA‐Tag (Figure 4A; Table S3; Section 2). Meanwhile, the full‐length scRNA‐seq data including 1200 cells from the same sample was obtained from our previously published study^32^ and five main cell types (tumour cell, lymphocyte, fibroblast, endothelial cell, and myeloid cell) were detected (Figure S3C). The scCPA‐Tag identified an absent epithelial cell cluster in scRNA‐seq, which may be due to the increased number of cells obtained. For the H3K27me3 profile data of scCPA‐Tag, we performed cell clustering by the cell‐feature matrix generated from fragments and assigned cell types by matching identical cell barcodes with those in chromatin accessibility data (Figure 4B,C). Notably, the cell membership within the H3K27me3 clusters corresponded highly (83.52%) to chromatin‐accessible clusters, indicating that the H3K27me3 profile can effectively identify various cell types in HCC (Figure 4C; Figure S3D).

For chromatin accessibility data of scCPA‐Tag, the cluster exhibiting high gene activity scores for *ALB*, *TTR*, *and APOA2* was identified as tumour cell (Figure 4D). Similarly, clusters with high gene activity scores of canonical cell type markers were identified as epithelial cell (*EPCAM*, *KRT19*), lymphocyte (*CD2*, *CD3D*, *MS4A1*), fibroblast (*PDGFRB*, *COL1A1*),^33^ endothelial cell (*PECAM1*, *VWF*, *CDH5*),^34^ and myeloid cell (*HLA‐DRA*, *C1QB*) (Figure 4D). The scRNA‐seq data from the same sample showed that these accessible marker genes were highly expressed in the corresponding cell types (Figure 4D). The H3K27me3 modification score of each gene was calculated using ArchR (Methods).^22^ As expected, we observed that repressive H3K27me3 modifications of most accessible marker genes were downregulated in the corresponding cell types (Figure 4D).

The GO enrichment analysis revealed that genes with lower H3K27me3 and higher accessibility in tumour cells were significantly enriched in small molecule metabolic process and lipid metabolic process pathways, etc. (Figure 4E). These pathways are linked to liver metabolism, aligning with the understanding that tumour cells originate from hepatocytes.^35^ In comparison, epithelial cells showed enrichment for the epithelium development pathway, suggesting their roles in promoting the growth and maintenance of epithelial tissues within the tumour microenvironment (Figure S3E). Endothelial cells exhibited enrichment in vasculature development and blood vessel morphogenesis pathways, indicating they may play a role in angiogenesis within the tumour microenvironment. Myeloid cells displayed enrichments in immune response and defense response pathways, suggesting that myeloid cells may engaged in eliminating tumour cells through immune surveillance (Figure S3E).

We also identified genes with high H3K27me3 modification in each cell type (Figure 4D). Most of these genes were related to other cell lineages, suggesting that H3K27me3 modification plays a role in regulating cell fate determination and lineage specification. For instance, in fibroblasts, T cell lineage markers *CD3E* and *CD2* exhibited high H3K27me3 modification (Figure 4D). Similarly, in tumour cells, we observed upregulation of H3K27me3 modification on immune cell lineage marker *PTPRC*, as well as olfactory receptor family members (*OR5M3, OR5M8*, and *OR13C4*), and homeobox proteins (*HOXB2, HOXA1*, and *HOXA4*), which are associated with sensory perception and embryonic development, respectively (Figure 4D; Figure S3F).^36^, ^37^ Moreover, we observed that immune‐related and cell adhesion‐related genes such as *CD83*, *HLA‐DRA*, *JAK3*, *CEACAM6*, and *ITGB2* ^38^, ^39^, ^40^, ^41^ exhibited high H3K27me3 modification in tumour cells, suggesting that their expression may be suppressed by H3K27me3 to facilitate immune escape of tumour cells (Figure S3F). Additionally, GO enrichment analysis of genes with high H3K27me3 modification in tumour cells showed enrichment in processes such as cell adhesion, lymphocyte activation, sensory perception, and embryonic skeletal system development (Figure 4F). These results indicate that tumour cells may exhibit high cell mobility and immune response suppression, and maintain a specific cell phenotype by H3K27me3 modification to inhibit genes of other cell lineages.

We performed motif enrichment analysis on chromatin‐accessible data of scCPA‐Tag and identified the TF regulators in each cell type (Figure S3G). By analyzing our previously published scRNA‐seq data from the same sample,^32^ we observed that target genes of enriched TFs also exhibited high expression in corresponding cell types. For instance, ELF2 and SP1, known for their roles in angiogenesis,^42^, ^43^ were enriched in endothelial cells, and their target genes were highly expressed in these cells (Figure S3G,H). Tumour cells showed enrichment of hepatocyte differentiation‐related TFs HNF4A/G and FOXA1/2,^44^ and highly expressed targeted genes of these TFs (Figure S3G,H). These results demonstrate a positive correlation between chromatin accessibility and gene expression patterns across different cell types in HCC.

In conclusion, scCPA‐Tag was utilized to analyze the heterogeneity of H3K27me3 modification and chromatin accessibility in various cell types in HCC. This approach provides a deeper understanding of the complex interplay between epigenetics and gene expression, as well as offering insights into the intricate processes involved in determining cell state or cell fate.

### 3.5 Histone modification and chromatin accessibility co‐regulated gene expression and TSS selection

To further investigate the epigenetic regulation of various cell types in HCC, we compared the epigenetic and transcriptome profiles in different cell types. In general, chromatin accessibility signals represented by gene activity score showed a positive correlation (*R* = 0.049–0.29, *p* < 0.05) with gene expression levels in all five cell types. However, we also observed that some genes (about average of 20.1%) in different cell types were not expressed even with high chromatin accessibility (Figure S4A). Most of these non‐expressed genes (more than 90%) could be explained by inhibition of H3K27me3 modification (Figure S4B). For example, the promoter region of *HES1* was accessible in all six cell types and specifically exhibited high H3K27me3 modification in lymphocytes (Figure 4G). These are reasonable to explain why *HES1*, a regulator in the differentiation decision of hemato‐endothelial fate,^45^ showed minimal expression specifically in lymphocytes but was highly expressed in tumour cells, fibroblasts, and especially endothelial cells (Figure 4H). Similarly, *HS6ST3* exhibited chromatin accessibility at its promoter region but lacked expression in endothelial cells, lymphocytes, and tumour cells due to the presence of H3K27me3 modification at its promoter region (Figure S4C,D).^46^ These results indicate that integration analysis of chromatin accessibility and histone modification provides a more comprehensive understanding of the mechanisms underlying gene regulation.

Besides, we also noticed that some genes showed expression even if high H3K27me3 modification was detected in their gene region. We speculated that scCPA‐Tag could provide valuable insights into the mechanisms underlying the selection of mRNA transcripts (alternative splicing, AS) for a gene. AS is a crucial process that allows a single gene to produce multiple protein isoforms with distinct functions.^47^ We observed the presence of multiple TSSs and 9 junctions, forming two AS forms (AS1: Junction 1−8, AS2: Junction 1−7 and Junction 9) for the *ACTA2* gene (Figure 4I,J). This result demonstrated a preference for AS2 in lymphocytes, while other cell types exhibited a predilection for AS1. (Figure 4I). *ACTA2* has been reported to be associated with the enhancement of hepatic stellate cell contractility, leading to liver fibrosis through the TGF‐β pathway.^48^, ^49^ According to the MANE (Matched Annotation between NCBI and EBI) Select,^50^ which includes a representative transcript for each protein‐coding locus, AS1 is considered the default transcript for *ACTA2*. The preferential selection of AS1 may be explained by its relatively low levels of H3K27me3 modification and its high chromatin accessibility at the TSS region of AS1, as demonstrated in fibroblasts (Figure 4I,J). In contrast, in lymphocytes, the expression of the AS2 transcript for *ACTA2* was observed instead of the AS1 transcript (Figure 4I). We found that H3K27me3 modification was more enriched at the TSS region of AS1 but lower at that of AS2 in lymphocytes. Correspondingly, the TSS of AS2 showed high chromatin accessibility compared with AS1 in lymphocytes (Figure 4J). These findings indicated that scCPA‐Tag can be applied to explain the selection mechanisms underlying multiple TSS sites.

In conclusion, scCPA‐Tag profiles not only provide a more accurate understanding of gene expression regulation mechanisms but also shed light on the mechanisms governing the selection of alternative transcripts.

### 3.6 Characterization of tumour cell subtypes by scCPA‐Tag

**FIGURE 5:**
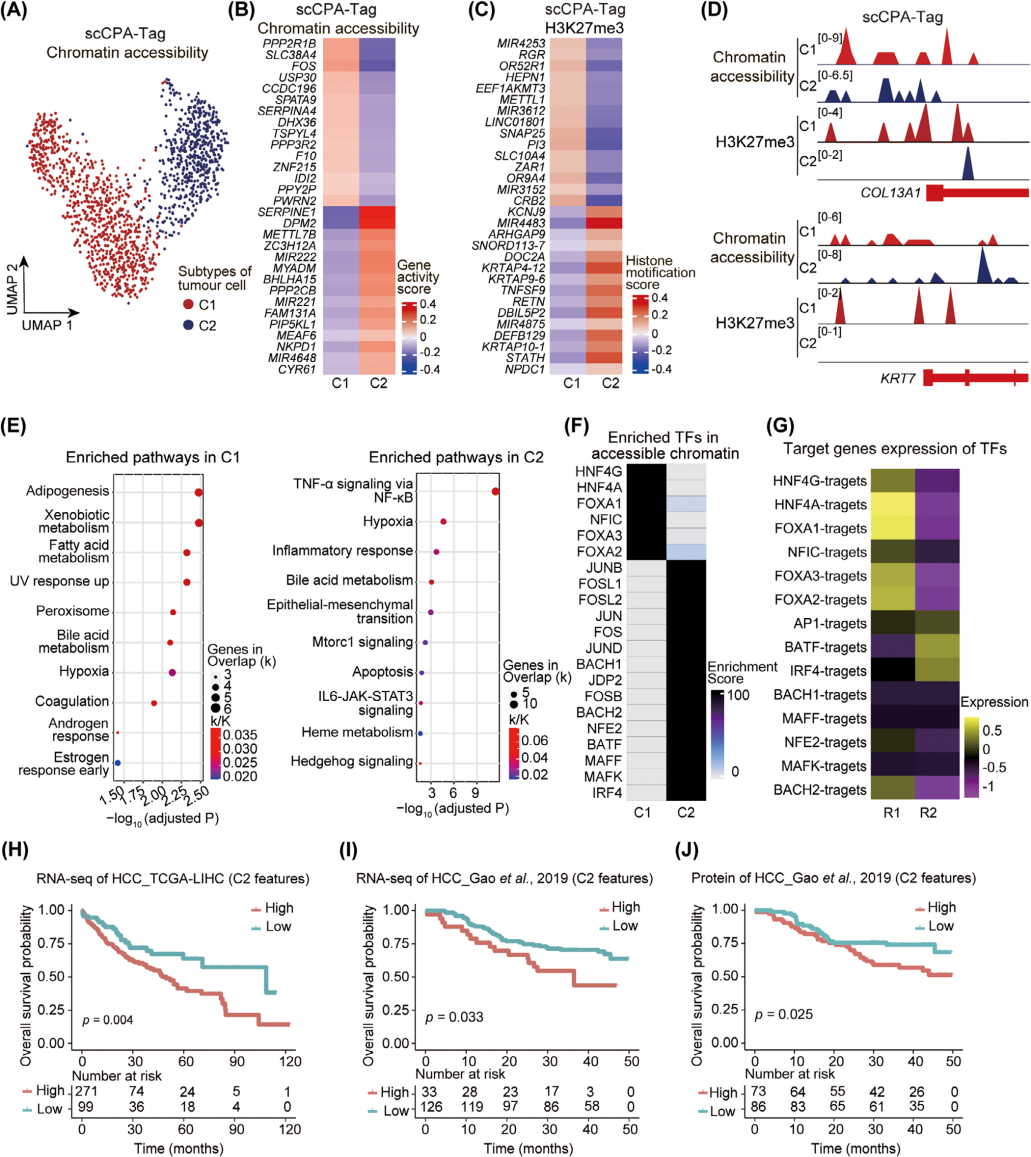
scCPA‐Tag reveals tumour cell heterogeneity. (A) The UMAP plot shows two subtypes of tumour cells clustered by chromatin accessibility profile generated by scCPA‐Tag. (B) The heatmap shows the specific chromatin accessibility genes in two tumour cell subtypes. (C) Heatmap shows the H3K27me3 modification upregulation genes in two tumour cell subtypes. (D) Genome browser tracks represent chromatin accessibility and H3K27me3 modification of *COL13A1* and *KRT7*. (E) The bubble plot shows the enriched pathways for chromatin accessibility data in the C1 cluster (left) and in the C2 cluster (right). (F) Heatmap shows the enriched TFs of chromatin‐accessible data in two tumour cell subtypes. (G) Heatmap shows expression of target genes of chromatin accessible data enriched TFs (in Figure 5F). (H) Kaplan–Meier curve shows the overall survival rate of patients, characterized by either low (cyan) or high (red) C2 features for RNA‐seq from the TCGA‐LIHC database. (I–J) Kaplan–Meier curve shows the overall survival rate of patients, characterized by either low (cyan) or high (red) C2 features for protein (I) and RNA‐seq (J) data from Gao et al.^74^ HCC dataset.

Investigating tumour cell heterogeneity holds immense significance in understanding disease progression and developing effective treatments. By analyzing chromatin‐accessible profiles obtained from scCPA‐Tag, we classified tumour cells into two distinct subtypes (Figure 5A). Cluster 1 (C1) exhibited high chromatin accessibility on tumour‐suppressive genes, such as *PPP2R1B*,^51^ *SLC38A4*,^52^ *SERPINA4*,^53^ and *DHX36*.^54^ Conversely, cluster 2 (C2) displayed high chromatin accessibility on genes associated with tumour promotion, including *SERPINE1*,^55^ *DMP2*,^56^ *MIR221*, *MIR222*,^57^ and *CYR61* ^58^, ^59^ (Figure 5B). *SERPINE1* exhibits pro‐angiogenic, growth‐promoting, migratory‐stimulating, and anti‐apoptotic properties, which collectively contributes to the promotion of tumour growth and metastasis.^55^ *MIR221* and *MIR222* were identified as regulators of epithelial‐to‐mesenchymal transformation (EMT) in breast cancer, contributing to the progression and metastasis of cancer.^57^ *CYR61*, an immediate early gene induced by growth factor, promotes angiogenesis and tumour growth.^58^ In addition, *CYR61* has been found to enhance the exosmosis of tumour cells, thereby promoting lung metastasis in breast cancer.^59^

The identification of tumour cell subtypes solely based on histone modification data was not feasible. However, through the alignment of identical cell barcodes, scCPA‐Tag allowed the identification of the distinct histone modification profiles of tumour cell subtypes, which provide additional epigenetic features to characterize the heterogeneity of tumour cells (Figure 5C). Genes such as *METTL1* ^60^ and *CRB2*,^61^ known to be associated with poor prognosis, showed upregulated H3K27me3 modification in C1, while tumour suppressor genes *ARHGAP9* ^62^, ^63^ and *TNFSF9* ^64^ showed upregulated H3K27me3 modification in C2 (Figure 5C). Furthermore, we observed that the genes associated with tumour poor prognosis, *KRT7* and *COL13A1*,^65^, ^66^, ^67^ exhibited chromatin accessibility in C1 and C2. However, H3K27me3 was located at these gene regions in C1 which may inhibit their expression (Figure 5D). In addition, the chromatin‐accessible profiles of C1 exhibited enrichment of pathways related to adipogenesis, fatty acid metabolism, and bile acid metabolism (Figure 5E). These pathways are associated with liver physiological metabolism functions.^68^ On the other hand, the pathways enriched in C2 included TNF‐α signalling via NF‐κB, inflammatory response, hypoxia, EMT, mTORC1 signalling, and IL6‐JAK‐STAT3 signalling, which were associated with tumour metastasis and growth (Figure 5E).^69^, ^70^, ^71^, ^72^ These results indicate that scCPA‐Tag enables simultaneously profiling the chromatin accessibility and histone modification (such as H3K27me3) in tumour cell subtypes, which provides potential therapeutic targets not only for inhibiting accessible genes but also for inducing genes with repressed modifications.

Based on our previously published scRNA‐seq data,^32^ tumour cells from HCC were separated into two clusters (R1 and R2). Genes with high chromatin accessibility in C1 enriched motifs for TFs including HNF4G, HNF4A, FOXA1, FOXA2, and FOXA3. R1 showed high expression of target genes for these enriched TF motifs (Figure 5F,G). Conversely, R2 highly expressed target genes of C2 accessible genes‐enriched motifs including AP‐1 (including Jun, JunB, JunD, FOS, FOSB, JDP2), IRF4, and BATF (Figure 5F,G). These results from scRNA‐seq provided validations for the tumour cell subtypes identification by scCPA‐Tag.

We further conducted a survival analysis to investigate the clinical significance of these epigenetic features of tumour cell subtypes in HCC. We explored the correlation between the high accessible genes (Table S4) of two tumour cell subtypes and overall survival (OS) in HCC patients from The Cancer Genome Atlas Liver Hepatocellular Carcinoma dataset (TCGA‐LIHC)^73^ and Gao et al.^74^ dataset. Our analysis revealed that high accessible genes of C1 were significantly associated with a favourable prognosis, while high accessible genes of C2 exhibited worse OS (*p* < 0.05) in both datasets (Figure 5H–J; Figure S5A–C). Collectively, we identified C2 as a more malignant subtype characterized by high chromatin accessibility and a lower H3K27me3 of feature genes associated with cell proliferation, invasion, and metastasis by scCPA‐Tag.

In summary, our study demonstrated the effective utilization of scCPA‐Tag to identify distinct tumour cell subtypes and revealed their epigenetic regulatory features. These results contribute to our understanding of cancer heterogeneity and may pave the way for developing targeted therapies tailored specifically for different subtypes based on their unique epigenetic profiles.

## 4 DISCUSSION

In this study, we developed a multi‐modal epigenetics method, scCPA‐Tag, which can simultaneously explore the histone modification/TF enrichment and chromatin‐accessible regions. scCPA‐Tag provides comprehensive epigenetic information that enables us to gain deeper insights into genomic characteristics and mechanisms of gene expression regulation. At present, several methods have been developed for analyzing multi‐dimensional epigenetic information, such as multiple histone modifications,^75^ DNA methylation with histone modification,^76^ and chromatin accessibility with histone modification.^9^, ^10^, ^11^ Among these techniques, CUT&Tag2for1 enables simultaneous profiling of the accessible and silenced regulome using an initiation form of RNA polymerase II (Pol2S5p) antibody and an H3K27me3 antibody.^9^ However, assigning Pol2S5p and H3K27me3 profiles generated from CUT&Tag2for1 based on the fragment size averages poses challenges in regions where the fragment distributions are similar or opposite.^9^ scGET‐seq^10^ and nano‐CT^11^ enable the capture of histone modification and chromatin accessibility in the same cell. scGET‐seq is limited to depicting only H3K9me3 modification through a hybrid molecule between Tn5 transposase and heterochromatin protein‐1α (HP‐1α) binding to H3K9me3 mark.^10^ nano‐CT can be applied to various histone modifications; however, the authors caution the potential increased nuclei loss and clumping.^11^ This could be attributed to cleaved DNA free from nuclei during prolonged antibody incubation. Nevertheless, in scCPA‐Tag, we did not observe the nuclei aggregation phenomenon. This could potentially be attributed to the fact that we performed the Tn5 transposition reaction after the antibody incubation to reduce the time after tagmentation. Furthermore, magnetic cell beads were employed in scCPA‐Tag, allowing for the recycling of beads after PCR. This enables the separation of amplified and sequenced accessible DNA and histone modification‐associated DNA, thereby reducing signal bias induced by PCR.

The scCPA‐Tag allows the investigation of the combined influence of chromatin accessibility and histone modification within the same cell, providing a more comprehensive epigenetic landscape. Relying solely on chromatin‐accessible data may not always yield accurate results when inferring gene expression.^8^ Notably, certain genes such as *PODXL* and *SAMD11* in the K562 cell line, as well as *HES1* in the HCC sample, were found to be unexpressed despite accessible chromatin (Figures 3C,D and 4G,H). This issue may be clarified by the inhibitory effect of H3K27me3 modification. In addition to elucidating gene expression regulations, scCPA‐Tag offers valuable insights into the mechanisms governing the selection of multiple TSSs. In the HCC sample, we observed alternative splicing events for *ACTA2* in different cell types attributed to the H3K27me3 modification on different TSSs. In conclusion, scCPA‐Tag provides dual epigenetic measurements that help clarify gene expression regulation and reveal the mechanisms governing the selection of multiple TSSs, highlighting the significance of considering both chromatin accessibility and histone modifications in epigenetic studies.

Furthermore, scCPA‐Tag enables to reveal heterogeneous cell populations in HCC. We observed that most genes with chromatin accessibility displayed reduced levels of H3K27me3 modification, while genes with upregulated H3K27me3 may be involved in maintaining their cell fate in different cell types. Finally, applying scCPA‐Tag to tumour cells revealed two distinct subtypes, including a malignant subtype C2 characterized by high chromatin accessibility and lower H3K27me3 in feature genes associated with proliferation, invasion, and metastasis, indicating a worse prognosis for HCC patients (Figure 5A–C,H). Recently, we have also applied scCPA‐Tag to study the dynamic alterations of epigenetic profiles during tumour metastasis colonization in HCC lung metastasis samples. By applying scCPA‐Tag in various samples, including the K562 cell line, human HCC sample, and HCC lung metastasis samples, we have demonstrated its broad applicability and utility.

The emergence of single‐cell multi‐modal techniques has facilitated the generation of vast amounts of single‐cell multi‐omics data. Integrating diverse dimensions of epigenetic information is crucial for a comprehensive understanding of gene regulatory mechanisms. However, systematically integrating these multi‐omics data remains a significant challenge. The development of bioinformatics methods to achieve multi‐omics data integration presents a promising and necessary direction for future research, driving discoveries and innovations in the field.

## 5 CONCLUSION

In conclusion, the scCPA‐Tag technique offers a novel and effective solution for investigating epigenetic regulation at the single‐cell level in developmental, differentiation, and disease processes. Our study demonstrated its applicability in revealing the complex epigenetic regulation in diverse cell types from HCC. Looking forward, this integrated approach will greatly enhance our understanding of intricate molecular mechanisms underlying chromatin regulation.

## AUTHOR CONTRIBUTIONS

Chunqing Wang, Liang Wu, and Yu Zhong conceived the study. Chunqing Wang, Liang Wu, Yu Zhong, Waidong Huang, and Xuanxuan Zou designed the experiments and analysis and wrote the manuscript. Waidong Huang and Chunqing Wang prepared the figures. Chunqing Wang optimized and performed the scCPA‐Tag experiments. Waidong Huang, Yu Zhong, Xuanxuan Zou, Shang Liu and Zihao Li optimized the analysis methodology and analyzed the data. Yunfan Sun and Kaiqian Zhou provided and prepared the HCC sample. Jie Li, Xi Chen, Jianhua Yin, Yinqi Bai reviewed and edited the manuscript. Shiping Liu, Xin Jin, Miaomiao Jiang, Chuanyu Liu and Longqi Liu provide resources supporting this study. Yaling Huang, Shanshan Wang, Yue Yuan, Qiuting Deng provided advice for the experiments of the study. Liang Wu and Xun Xu supervised the project.

## CONFLICT OF INTEREST STATEMENT

The authors declare no conflict of interest.

## ETHICS STATEMENT

The patient gave informed consent for the collection of clinical information, tissue collection, and research testing under Institutional Review Board (IRB)‐approved protocols (B2021‐848‐R) at Zhongshan Hospital Fudan University. The single‐cell experiments of the K562 cell line and clinical sample obtained approval from the Institutional Review Board of BGI (BGI‐IRB 22163‐T1).

## Supporting information

Supporting Information

Supporting Information

Supporting Information

Supporting Information

Supporting Information

Supporting Information

## Data Availability

The original data of scCUT&Tag, snATAC‐seq, and scCPA‐Tag that support the findings of this study have been deposited into the CNGB Sequence Archive (CNSA)^77^ of China National GeneBank DataBase (CNGBdb)^78^ with accession number CNP0003538. The scRNA‐seq data of the HCC sample was downloaded from CNGBdb, CNP0000650, and the scRNA‐seq data of K562 cells was obtained from CNP0004330.
