# Supplementary figures and images for: Single‐cell multi‐modal chromatin profiles revealing epigenetic regulations of cells in hepatocellular carcinoma
Source: Clin Transl Med. 2024 Aug 29;14(9):e70000. doi: 10.1002/ctm2.70000 (PMC11362026; doi:10.1002/ctm2.70000)

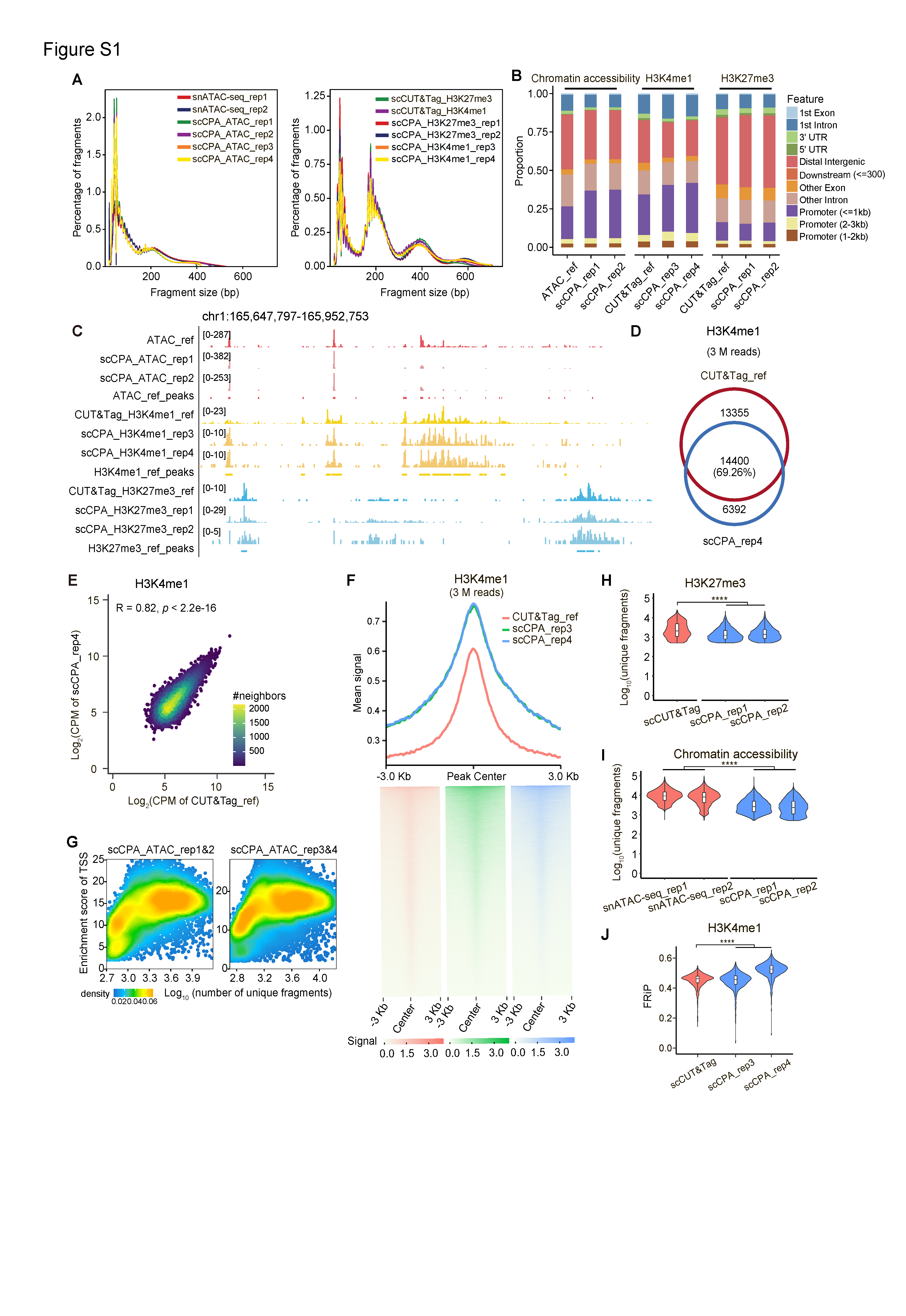

Supplement: Supplementary file 1 — Supporting Information [file CTM2-14-e70000-s003.tif]

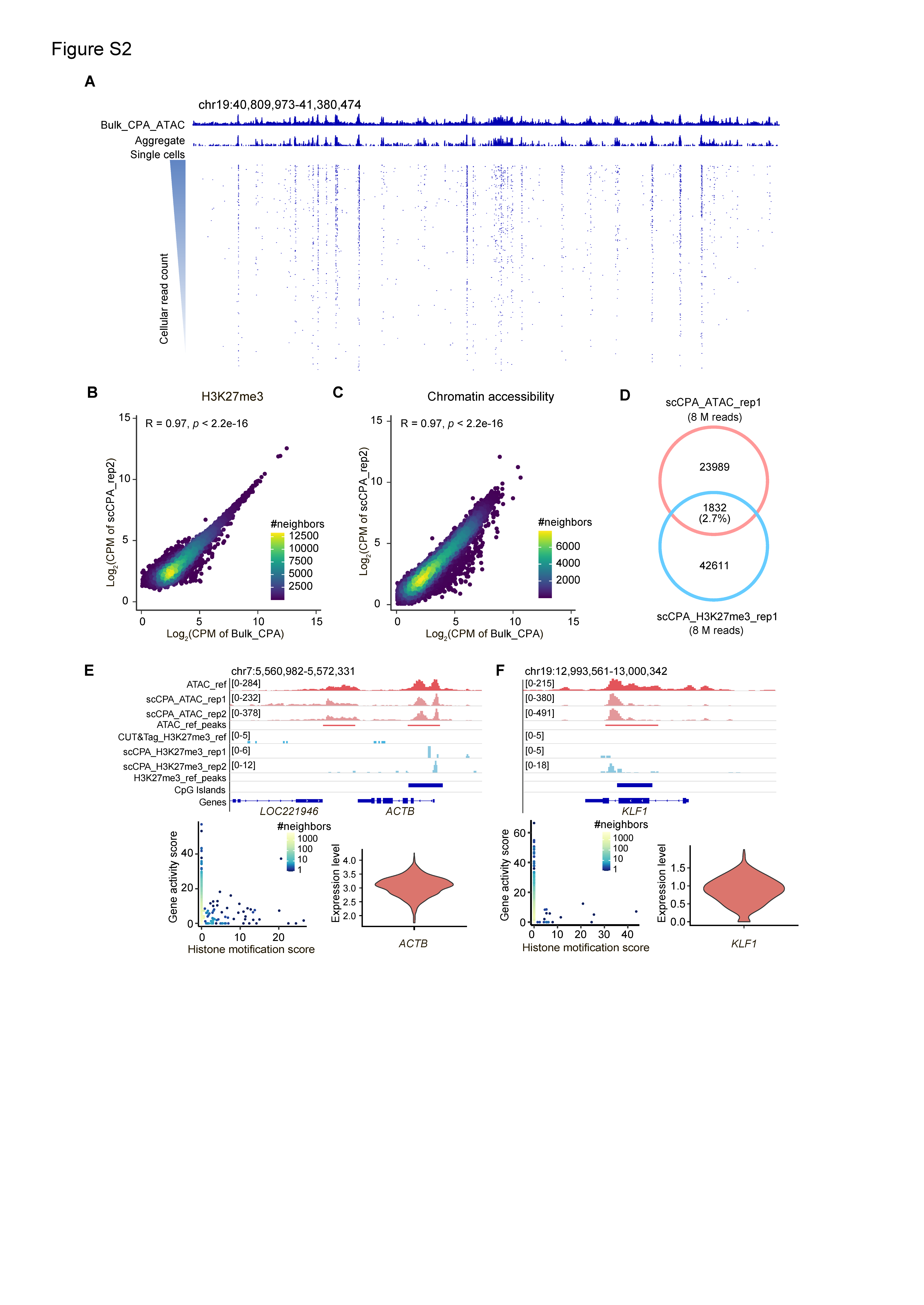

Supplement: Supplementary file 2 — Supporting Information [file CTM2-14-e70000-s001.tif]

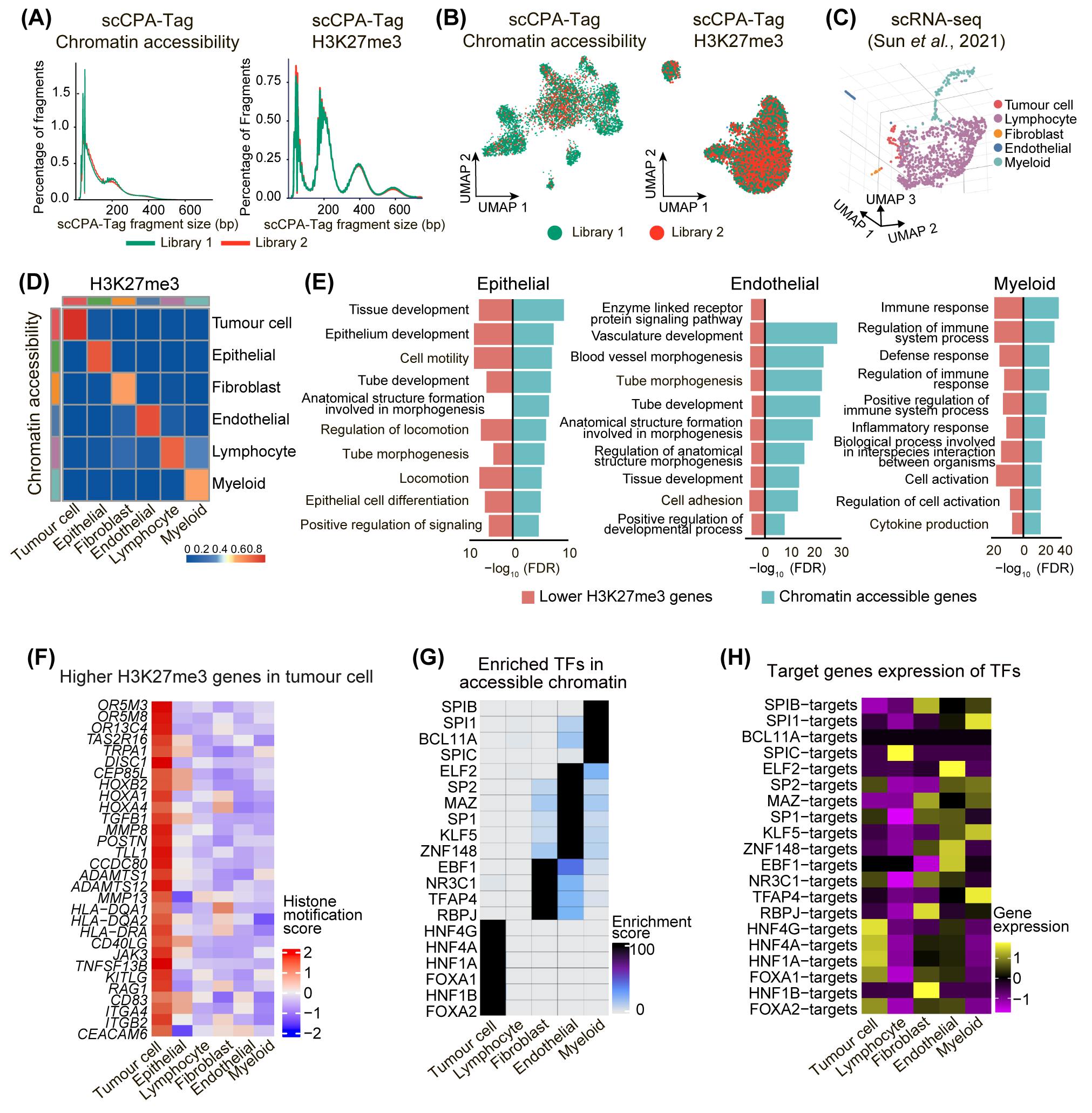

Supplement: Supplementary file 3 — Supporting Information [file CTM2-14-e70000-s005.tif]

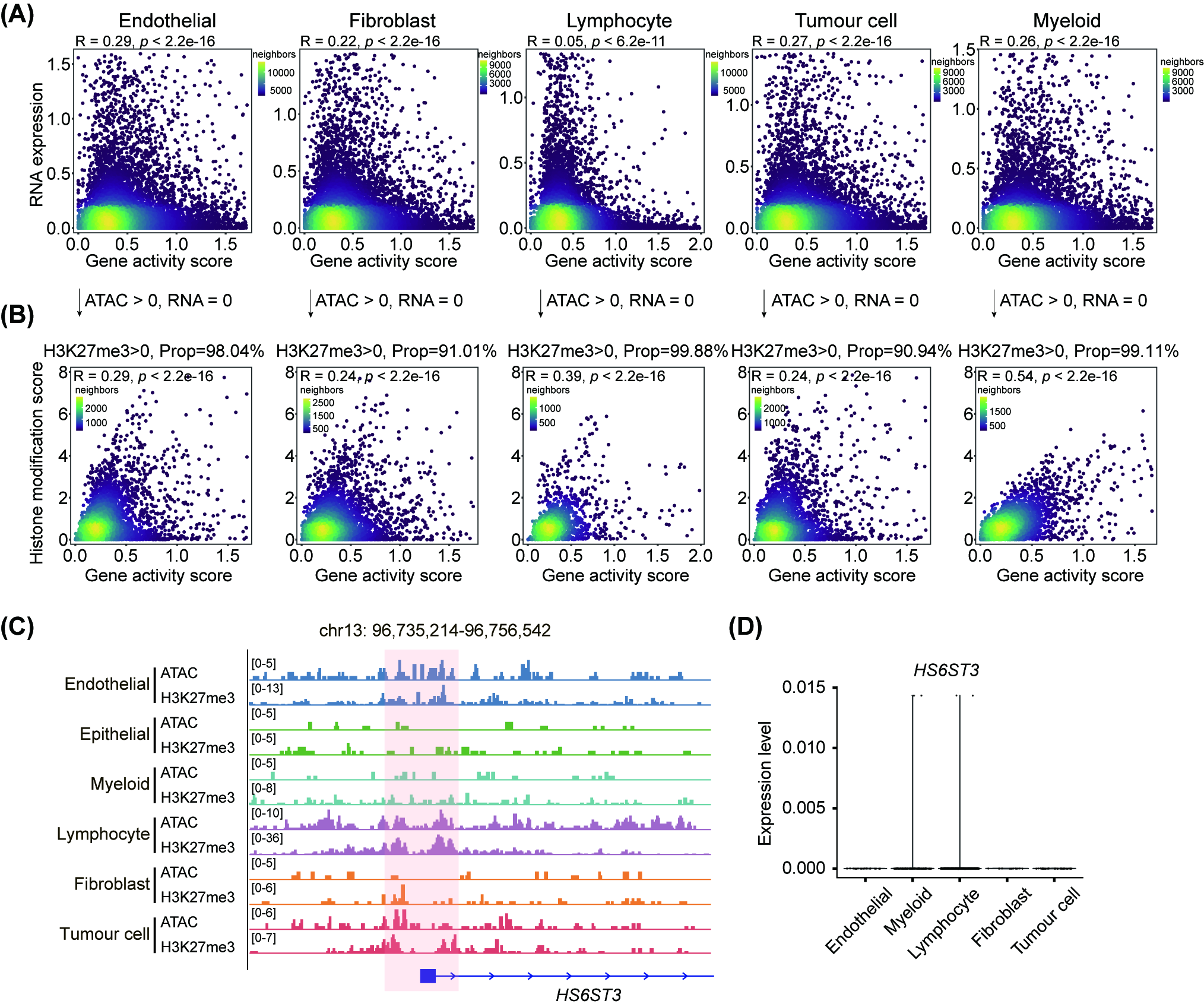

Supplement: Supplementary file 4 — Supporting Information [file CTM2-14-e70000-s006.tif]

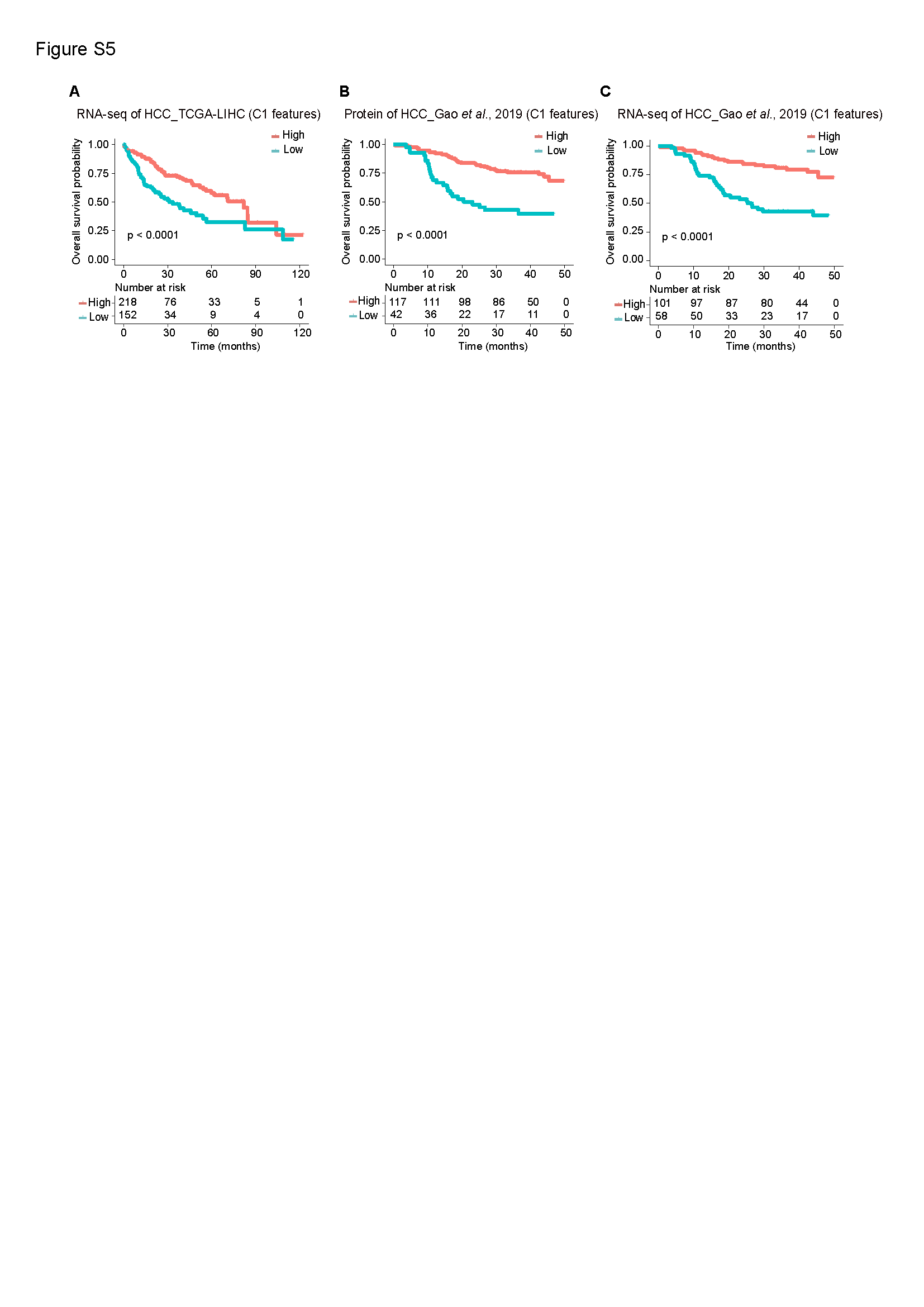

Supplement: Supplementary file 5 — Supporting Information [file CTM2-14-e70000-s004.tif]
